# *Aristathlus imperatorius* Bergroth, a newly recognized synonym of *Reduvius iopterus* Perty, with the new combination *Aristathlus iopterus* (Perty, 1834) (Hemiptera: Reduviidae: Harpactorinae) ​

**DOI:** 10.3897/BDJ.3.e5152

**Published:** 2015-06-16

**Authors:** Hélcio R. Gil-Santana, Dimitri Forero

**Affiliations:** ‡ Laboratório de Diptera, Instituto Oswaldo Cruz, Av. Brasil, 4365, 21040-360, Rio de Janeiro, Brazil; § Departamento de Biología, Laboratorio de Entomología, UNESIS, Pontificia Universidad Javeriana, Carrera 7 #43-82, Bogotá, Colombia

**Keywords:** Heteroptera, Harpactorini, Neotropical Region, new combination, new synonym

## Abstract

**Background:**

*Reduvius iopterus* Perty 1834 was first described from the Rio Negro, Brazil. It was then included in *Zelus* Fabricius 1803 as *Z. iopterus* (Perty 1834) without critical examination of this action (Stål 1872).The Neotropical genus *Aristathlus* Bergroth 1913 was described to include two species from the Amazon basin: *A. imperatorius* Bergroth 1913 and *A. regalis* Bergroth 1913.

**New information:**

Based on the original description and illustration of *Reduvius iopterus*, *Aristathlus imperatorius* is considered to be its junior synonym, with the resulting new combination: *Aristathlus iopterus* (Perty 1834).

## Introduction

Upon description by Bergroth (1913), the genus *Aristathlus* included two species: *A. imperatorius* Bergroth 1913 (type species of the genus by original designation) and *A. regalis* Bergroth 1913. Forero et al. (2008) revised the genus *Aristathlus* and provided redescriptions and diagnoses for the species, as well as detailed documentation of the male genitalia.

While Bergroth (1913) regarded *Aristathlus* as close to *Zelus*, Forero et al. (2008) provided notes comparing these two genera with each other and also with another four Neotropical harpactorine genera with elongate bodies and heads (*Atopozelus* Elkins 1954, *Ischnoclopius* Stål 1868, *Iquitozelus* Bérenger 2003, and *Heza* Amyot and Serville 1843). Forero et al. (2008) concluded that the species of *Aristathlus* do not belong in these genera because of the particular structure of the head, legs and genitalia.

*Aristathlus imperatorius*, originally described from French Guiana, has been recorded from Brazil (states of Amazonas, Pará, and Mato Grosso) (Gil-Santana 2007), Colombia, and Peru (Forero et al. 2008). This species shows variation in pronotal color pattern, ranging from a narrow, median U-shaped pale or yellowish marking, to a wider marking reaching the humeral angles (Gil-Santana 2007), as well as the metanotum, which is yellow or sometimes black (Forero et al. 2008).

**Figure 1.**
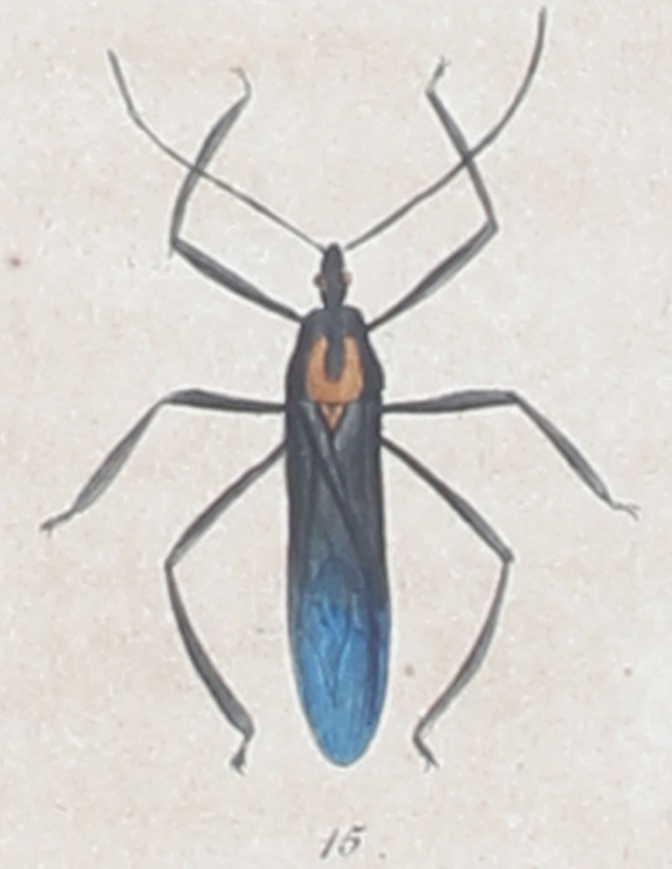
Dorsal habitus of *Reduvius iopterus* as illustrated by Perty (1834) (his figure 15, plate XXXIV).

*Reduvius iopterus* Perty, 1834 was described from the region of the Rio Negro (a river in the Amazon region of Brazil), and its original description was accompanied by a dorsal habitus drawing (Perty 1834) (Fig. 1). Stål (1872) included this species in the genus *Zelus* Fabricius, 1803, and since then, it has been listed in catalogues *as Z. iopterus* (Perty 1834) (Lethierry and Severin 1896, Wygodzinsky 1949​, Maldonado C. 1990).

The type specimen of *Reduvius iopterus* ought to be deposited at the Zoological Collection in Munich, as are many of Perty's type specimens, but it is now considered lost (Burmeister 1983). Forero et al. (2008) were also unable to locate the type specimens of *A. imperatorius*.

## Materials and methods

The depository institutions of the studied material are the following: American Museum of Natural History, New York (AMNH), Instituto de Ciencias Naturales, Universidad Nacional de Colombia, Bogotá, Colombia (ICN), Universidade Federal do Rio de Janeiro, Brazil (MNRJ).

## Taxon treatments

### Aristathlus iopterus

(Perty, 1834) new combination

Reduvius iopterus Perty, 1834: Perty (1834)
Aristathlus imperatorius Bergroth, 1913: Bergroth (1913)** new synonym**

#### Materials

- **Type status:** Other material. **Occurrence:** recordedBy: Elias & Roppa; individualCount: 1; sex: male; lifeStage: adult; **Taxon:** taxonID: urn:lsid:organismnames.com:name:4352608; scientificName: *Aristathlus imperatorius* Bergroth, 1913; kingdom: Animalia; phylum: Arthropoda; class: Insecta; order: Hemiptera; family: Reduviidae; genus: Aristathlus; specificEpithet: imperatorius; scientificNameAuthorship: Bergroth, 1913; **Location:** country: Brazil; countryCode: BR; stateProvince: Amazonas; locality: Manaus; **Identification:** identifiedBy: Hélcio R. Gil-Santana; dateIdentified: 2007; **Event:** samplingProtocol: none specified; eventDate: 1955-11-14; **Record Level:** modified: 2015-04-14; language: en; institutionCode: MNRJ; collectionCode: Entomology; basisOfRecord: PreservedSpecimen
- **Type status:** Other material. **Occurrence:** recordedBy: F. Oliveira; individualCount: 1; sex: female; lifeStage: adult; **Taxon:** taxonID: urn:lsid:organismnames.com:name:4352608; scientificName: *Aristathlus imperatorius* Bergroth, 1913; kingdom: Animalia; phylum: Arthropoda; class: Insecta; order: Hemiptera; family: Reduviidae; genus: Aristathlus; specificEpithet: imperatorius; scientificNameAuthorship: Bergroth, 1913; **Location:** country: Brazil; countryCode: BR; stateProvince: Pará; locality: Óbidos; **Identification:** identifiedBy: Hélcio R. Gil-Santana; dateIdentified: 2007; **Event:** samplingProtocol: none specified; eventDate: 1956-05; **Record Level:** modified: 2015-04-14; language: en; institutionCode: MNRJ; collectionCode: Campos Seabra collection; basisOfRecord: PreservedSpecimen
- **Type status:** Other material. **Occurrence:** recordedBy: E. Furtado; individualCount: 1; sex: male; lifeStage: adult; **Taxon:** taxonID: urn:lsid:organismnames.com:name:4352608; scientificName: *Aristathlus imperatorius* Bergroth, 1913; kingdom: Animalia; phylum: Arthropoda; class: Insecta; order: Hemiptera; family: Reduviidae; genus: Aristathlus; specificEpithet: imperatorius; scientificNameAuthorship: Bergroth, 1913; **Location:** country: Brazil; countryCode: BR; stateProvince: Mato Grosso; locality: Diamantino, Arinos River; verbatimLatitude: 14° 22' S; verbatimLongitude: 56° 07' W; **Identification:** identifiedBy: Hélcio R. Gil-Santana; dateIdentified: 2007; **Event:** samplingProtocol: none specified; eventDate: 2002-11-16; **Record Level:** modified: 2015-04-14; language: en; institutionCode: MNRJ; collectionCode: Entomology; basisOfRecord: PreservedSpecimen
- **Type status:** Other material. **Occurrence:** recordedBy: Mus. Goeldi 17/10 D01; individualCount: 1; sex: female; lifeStage: adult; **Taxon:** taxonID: urn:lsid:organismnames.com:name:4352608; scientificName: *Aristathlus imperatorius* Bergroth, 1913; kingdom: Animalia; phylum: Arthropoda; class: Insecta; order: Hemiptera; family: Reduviidae; genus: Aristathlus; specificEpithet: imperatorius; scientificNameAuthorship: Bergroth, 1913; **Location:** country: Brazil; countryCode: BR; locality: no specific locality; **Identification:** identifiedBy: P. Wygodzsinky; **Event:** samplingProtocol: none specified; **Record Level:** modified: 2015-04-14; language: en; institutionCode: AMNH; collectionCode: Entomology; basisOfRecord: PreservedSpecimen
- **Type status:** Other material. **Occurrence:** recordedBy: L. Richter; individualCount: 1; sex: male; lifeStage: adult; **Taxon:** taxonID: urn:lsid:organismnames.com:name:4352608; scientificName: *Aristathlus imperatorius* Bergroth, 1913; kingdom: Animalia; phylum: Arthropoda; class: Insecta; order: Hemiptera; family: Reduviidae; genus: Aristathlus; specificEpithet: imperatorius; scientificNameAuthorship: Bergroth, 1913; **Location:** country: Colombia; countryCode: CO; stateProvince: Meta; locality: Rio Ocoa; minimumElevationInMeters: 100; maximumElevationInMeters: 100; **Identification:** identifiedBy: D. Forero; dateIdentified: 2008; **Event:** samplingProtocol: none specified; eventDate: 1945-05-20; habitat: selva; **Record Level:** modified: 2015-04-14; language: en; institutionCode: ICN; collectionCode: Entomology; basisOfRecord: PreservedSpecimen
- **Type status:** Other material. **Occurrence:** recordedBy: E.L. Schlinger & E.S. Ross; individualCount: 1; sex: male; lifeStage: adult; **Taxon:** taxonID: urn:lsid:organismnames.com:name:4352608; scientificName: *Aristathlus imperatorius* Bergroth, 1913; kingdom: Animalia; phylum: Arthropoda; class: Insecta; order: Hemiptera; family: Reduviidae; genus: Aristathlus; specificEpithet: imperatorius; scientificNameAuthorship: Bergroth, 1913; **Location:** country: Peru; countryCode: PE; stateProvince: Huanuco; locality: Tingo María, Monzón Valley; **Identification:** identifiedBy: P. Wygodzsinky; **Event:** samplingProtocol: none specified; eventDate: 1954-09-23; **Record Level:** modified: 2015-04-14; language: en; institutionCode: AMNH; collectionCode: Entomology; basisOfRecord: PreservedSpecimen

#### Taxon discussion

**Figure 2.**
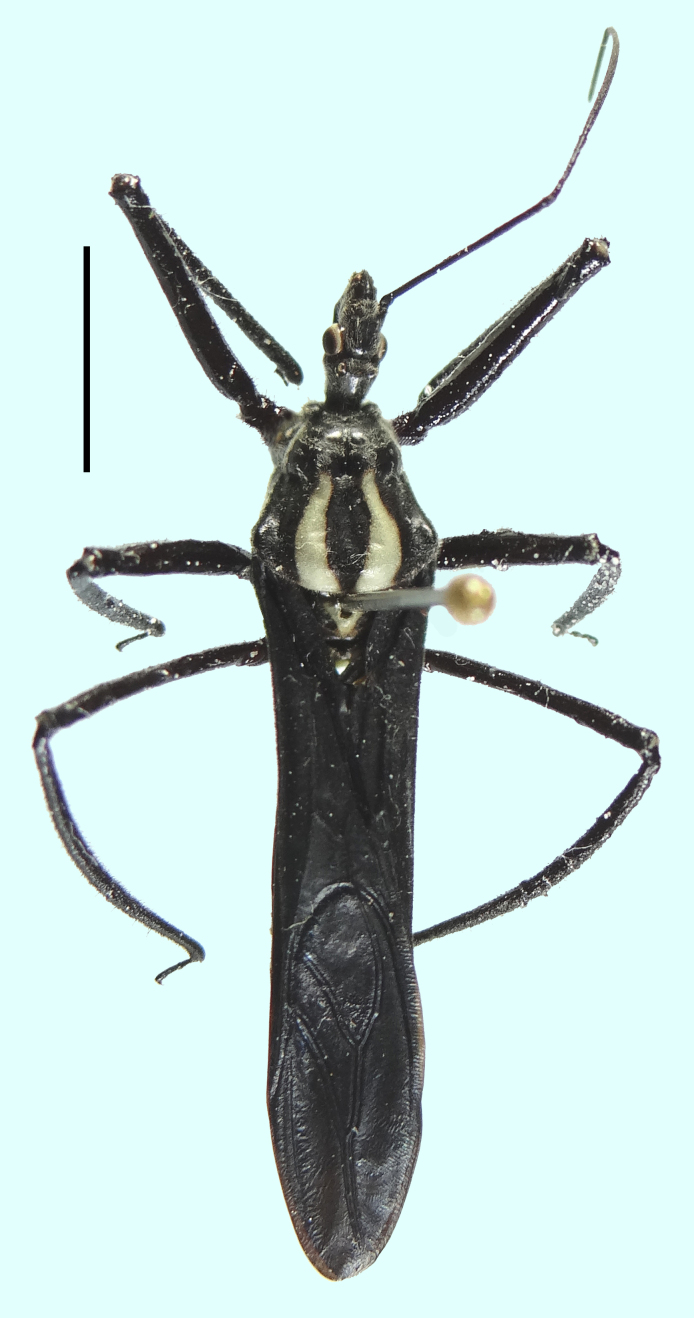
Dorsal habitus of *Aristathlus iopterus* (Perty 1834), male, [Brazil, Amazonas, Manaus]. Scale bar 5 mm.

Given that Perty’s type specimen is lost, we base our conclusions on Perty's original description and illustration of *R. iopterus* (Perty 1834) (Fig. 1). We also examined several specimens identified as *A. imperatorius* (Fig. 2, Gil-Santana 2007 and Forero et al. 2008). The examined material is deposited in the following institutions: American Museum of Natural History, New York, USA (AMNH), Instituto de Ciencias Naturales, Universidad Nacional de Colombia, Bogotá, Colombia (ICN), Universidade Federal do Rio de Janeiro, Brazil (MNRJ). From our examination of the specimens of *A. imperatorius*, we conclude that this species is a junior subjective synonym of *Reduvius iopterus*.

Both Perty (1834) and Bergroth (1913) described or illustrated the same color pattern and body structure for both *R. iopterus* and *A. imperatorius*. The main congruent aspects are the pale or yellowish U-like pattern on the dark posterior lobe of the pronotum, the yellow scutellum, the dark hemelytra, the metallic colored hemelytral membrane, and the swollen mesofemur (Forero et al. 2008) (Figs 1, 2).

There is no doubt that a synonymic proposal would be better based on the examination of the type specimens of the taxa involved. However, the identities of the known species in *Aristathlus* are not in dispute, even though Bergroth’s type specimens for both species have not been located and may be lost (Forero et al. 2008).

Neotype designations have to be done carefully (Winston 1999). Article 75 of the International Code of Zoological Nomenclature (ICZN 1999) clearly states that if there is no complex problem to be resolved (such as the identity of a taxon), there should not be any neotype designations (Article 75.2). Because the identity of *Aristathlus* species is not in dispute, we therefore refrain from designating such neotypes.

The taxonomic revision was carried out by accurately examining the differences between male gentalia of the two species involved. These differences are congruent with the color patterns exhibited by each of the species. Therefore, color pattern is an accurate way of identifying species in *Aristathlus* even when no other morphological data are available, which is the situation faced here. As far as we are aware, there are no species of Neotropical Harpactorini that could be confused with *R. iopterus* or *A. imperatorius*, based either on the coloration pattern or on the body and leg structure. *Reduvius iopterus* cannot be confused with the other species of *Aristathlus*, *A. regalis*, since it is easily distinguished from *R. iopterus* or *A. imperatorius* by its conspicuous yellow markings, including a transverse yellow band on the corium (Forero et al. 2008​). We regard the evidence presented sufficient to warrant the synonym proposed here.

## Discussion

Even though we have not examined the type specimens for these two species, the original descriptions (Bergroth 1913, Perty 1834) and the illustration from (Perty 1834) (Fig. 1) allow us to confidently propose the synonymy suggested above, given that the color pattern and leg structure are the same in the two named species.

It is also noteworthy that both taxa were recorded from the Amazon region, in which there is no other species known to us with the same general coloration and body structure, which reinforces the present synonymy. The superficial resemblance of *Aristathlus* to *Zelus* in the elongated body, as suggested by Bergroth (1913), was possibly the reason why *Reduvius iopterus* was placed in *Zelus* (as *Zelus iopterus*) by Stål (1872).

Finally, in accordance with Article 67 of the International Code of Zoological Nomenclature (ICZN 1999), *A. imperatorius* Bergroth, a junior synonym of *A. iopterus* (Perty) still remains the type species of *Aristathlus* Bergroth.

## Supplementary Material

XML Treatment for Aristathlus iopterus
